# Guided and unguided internet-delivered psychodynamic therapy for social anxiety disorder: A randomized controlled trial

**DOI:** 10.1038/s44184-024-00063-0

**Published:** 2024-05-10

**Authors:** Jakob Mechler, Karin Lindqvist, Kristoffer Magnusson, Adrián Ringström, Johan Daun Krafman, Pär Alvinzi, Love Kassius, Josefine Sowa, Gerhard Andersson, Per Carlbring

**Affiliations:** 1 Department of Psychology, Stockholm University, Stockholm, Sweden; 2 Centre for Psychiatry Research, Department of Clinical Neuroscience, Karolinska Institutet, & Stockholm Health Care Services, Region Stockholm, Stockolm, Sweden; 3 Department of Psychology, Uppsala University, Uppsala, Sweden; 4 Department of Behavioural Sciences and Learning, Linköping University, Linköping, Sweden; 5 Department of Clinical Neuroscience, Karolinska Institute, Stockolm, Sweden

**Keywords:** Outcomes research, Anxiety

## Abstract

Social Anxiety Disorder (SAD) is highly prevalent and debilitating disorder. Treatments exist but are not accessible and/or helpful for all patients, indicating a need for accessible treatment alternatives. The aim of the present trial was to evaluate internet-delivered psychodynamic therapy (IPDT) with and without therapist guidance, compared to a waitlist control condition, in the treatment of adults with SAD. In this randomized, clinical trial, we tested whether IPDT was superior to a waitlist control, and whether IPDT with therapeutic guidance was superior to unguided IPDT. Participants were recruited nationwide in Sweden. Eligible participants were ≥ 18 years old and scoring ≥ 60 on the Liebowitz Social Anxiety Scale self-report (LSAS-SR) whilst not fulfilling any of the exclusion criteria. Included participants were randomly assigned to IPDT with guidance (*n* = 60), IPDT without guidance (*n* = 61), or waitlist (*n* = 60). The IPDT intervention comprised eight self-help modules based on affect-focused dynamic therapy, delivered over 8 weeks on a secure online platform. The primary outcome was SAD symptoms severity measured weekly by the LSAS-SR. Primary analyses were calculated on an intention-to-treat sample including all participants randomly assigned. Secondary outcomes were depressive symptoms, generalized anxiety, quality of life, emotion regulation and defensive functioning. At post-treatment, both active treatments were superior to the waitlist condition with guided treatment exhibiting larger between group effects than unguided treatment (*d* = 1.07 95% CI [0.72, 1.43], *p* < .001 and *d* = 0.61, 95% CI [0.25, 0.98], *p* = .0018) on the LSAS-SR respectively. Guided IPDT lead to larger improvements than unguided IPDT (*d* = 0.46, 95% CI [0.11, 0.80], *p* < .01). At post-treatment, guided IPDT was superior to waitlist on all secondary outcome measures. Unguided IPDT was superior to waitlist on depressive symptoms and general anxiety, but not on emotion regulation, self-compassion or quality of life. Guided IPDT was superior to unguided PDT on depressive symptoms, with a trend towards superiority on a measure of generalized anxiety. At six and twelve month follow-up there were no significant differences between guided and unguided IPDT. In conclusion, IPDT shows promising effects in the treatment of SAD, with larger benefits from guided IPDT compared to non-guided, at least at post-treatment. This finding increases the range of accessible and effective treatment alternatives for adults suffering from SAD. The study was prospectively registered at ClinicalTrials (NCT05015166).

## Introduction

Social anxiety disorder (SAD) is characterized by a marked fear/anxiety or avoidance of social interactions as well as situations in which one is under scrutiny and/or the center of attention. Individuals suffering from SAD fear being negatively evaluated by others, humiliating themselves, being embarrassed, rejected or of offending others. To receive a diagnosis of SAD, this fear should also be out of proportion^1^. SAD is a prevalent and disabling disorder with a lifetime prevalence of 4% globally. Although prevalence varies considerably between different countries, data suggests that SAD is a common mental health condition that typically debuts early in life and tends to persist over time^2^. Furthermore, research suggests that the COVID–19 pandemic has led to elevated symptoms of social anxiety and that social distancing contributed to the maintenance of symptoms of social anxiety for many individuals^3,4^.

Fortunately, both psychotherapy and pharmacotherapy have proven to be effective in the treatment of SAD^5^, with Cognitive Behavioral Therapy (CBT) often being described as the gold standard treatment^6^. Numerous randomized controlled trials (RCTs) exist supporting the efficacy of CBT in the treatment of SAD^7^, with effects seeming to be maintained over time^8,9^. There are some indications that effects of CBT in SAD has been overestimated since higher quality studies have been associated with smaller effects^7^.

Although effective treatments exist, many patients do not gain access to and/or utilize them. Research suggests that anxiety disorders worldwide are underdiagnosed and undertreated. It has been estimated that, in a given year, less than 10% of individuals with anxiety disorders are receiving adequate treatment. Low-income countries have a wider treatment gap, indicating that individuals with anxiety disorders in these countries are less likely to receive treatment compared to those in more affluent countries^10^. Some research suggests that patients with SAD are less likely to utilize mental health treatment compared to patients suffering from mood disorders or panic disorder^11^. This is concerning, as SAD is associated with decreased quality of life^12^ and higher societal and personal costs compared to healthy controls^13^. In addition to treatment barriers related to limited access to treatment, research also suggest that individuals with SAD refrain from seeking treatment due to shame and perceived stigma – which is also in line with the core symptoms of the disorder. Logistical and financial barriers are also described as reasons for not seeking available treatment^14^. Developing treatment alternatives that are flexible regarding time and delivery of treatment, cost-effective and less reliant on face-to-face contact could therefore increase the number of patients with SAD receiving adequate treatment. It has been suggested that treatments delivered over the internet can overcome several of the aforementioned barriers to seeking and receiving treatment^15^.

CBT for SAD has been successfully tested delivered through the internet (ICBT) with results suggesting that it is non-inferior to CBT delivered in a face-to-face group format^16^. A recent meta-analysis found that ICBT was effective in comparison to various control groups and that it was equally efficacious as CBT delivered face-to-face^17^. Since then, another meta-analysis corroborated these findings, and different forms of digitally delivered CBT was also found to be more effective than other, non-CBT, digitally delivered treatments^18^. Although highly effective, conventional CBT is not associated with beneficial outcome for everyone entering treatment, it seems that roughly 40–50% of patients do not respond adequately^19^. Leichsenring and Leweke^20^ found that up to 40% of SAD sufferers did not achieve remission, even when receiving state-of-the-art treatments. In ICBT, when adhering to the criteria stipulated by Jacobson and Truax^21^, 38–56% of patients seem to achieve clinically significant change^22^. Since most patients tend to prefer psychological treatment before medication, it has been argued that alternatives to CBT need to be developed and tested^23^. One such alternative is psychodynamic psychotherapy (PDT). Overall, PDT seems to be effective in the treatment of anxiety disorders, but studies are still relatively few and of varying quality^24^. In terms of studies assessing the efficacy of PDT targeting SAD, delivered individually or in group, 13 RCTs exist^25^. Although of varying quality, most of these studies support the effectiveness of PDT in the treatment of SAD to some extent. Especially noteworthy are the studies comparing individual PDT to CBT. Leichsenring et al.^26^ conducted a large, multicenter RCT *(n* = 495), where both treatments were found more effective than a waitlist condition at post-assessment. PDT did not differ significantly from CBT regarding the number of responders, but significantly more patients remitted in CBT. However, this effect was small (*h* = 0.22), and no longer present at follow-up^27^. A latter study found highly similar results comparing the exact same treatments in adolescent suffering from SAD^28^. Bögels et al.^29^ compared PDT to CBT in adults with SAD, showing large within-group effects and high rates of remission, with no significant differences between treatments at the end of treatment or at follow-up.

Recently, PDT has also been adapted to being delivered over the internet (IPDT). Although relatively few studies exist, results are promising^30^. IPDT has been found to be non-inferior compared to ICBT in the treatment of adolescent depression^31^, with significant effects on comorbid anxiety as well. Johansson et al.^32^ showed that a ten-week IPDT treatment was significantly superior to waitlist (*d* = 1.05) for patients with SAD, with effects maintained over a two-year follow-up. Most IPDT interventions have been in the format of guided self-help^30^, and the effect of unguided IPDT (i.e., pure self-help without therapeutic support) is yet to be tested.

The aim of this study was to evaluate the efficacy of an IPDT programme, with and without therapeutic guidance, for patients with SAD, with SAD symptoms as primary outcome. Secondary outcomes included comorbid depression, generalized anxiety, dysregulated emotions, quality of life and defensive functioning. We hypothesized that (1) both guided and unguided IPDT would lead to a reduction in social anxiety compared to a waitlist control condition; (2) guided IPDT would be superior compared to unguided IPDT at post-treatment; (3) both IPDT treatments would be significantly superior to waitlist on secondary outcomes; and (4) guided IPDT would superior to unguided IPDT on secondary outcomes.

## Methods

In this randomized controlled trial, eligible participants were randomly allocated to guided IPDT, unguided IPDT or waitlist with an allocation ratio of 1:1:1. The study was approved by the Swedish Ethical Review Authority, reference number 2021-00026/03068. The study was prospectively registered at ClinicalTrials (NCT05015166).

### Participants

Participants were recruited nationwide in Sweden between August and September 2021 through advertisements on social media as well as relevant discussion forums on the internet. Interested participants were referred to a study website with information about the study and the treatment format (i.e., guided/unguided self-help, waitlist, duration and intensity of treatment). Those interested in partaking could then start the application process via a link on the website.

Inclusion criteria were: adults (≥18 years) presenting with a primary diagnosis of SAD confirmed by scoring ≥ 60 on the LSAS-SR, having access to a computer/smartphone/tablet with internet connection, and being able to read, write, and speak Swedish. Participants were excluded if they fulfilled any of the following exclusion criteria: scores ≤ 59 on LSAS-SR, substantial risk of suicide (i.e., clear intent and/or plans) and/or suicide attempts in the last three months as assessed by the Columbia-Suicide Severity Rating Scale Self-report (C-SSRS^33^), primary diagnosis of severe major depression (scores of ≥ 20 on Patient Health Questionnaire [PHQ-9^34^]), on-going participation in other psychological treatment(s), and psychotropic medication not stable the last month or with planned adjustments within the coming three months. Participants were also excluded if they fulfilled a primary diagnosis other than SAD as expressed in their applications to the research project. Participants diagnosed with autism spectrum disorders were also excluded. Withdrawal from the study was considered for participants who expressed increased suicidality; if deemed necessary they were referred to psychiatric services. Written informed consent was provided by participants online before screening. Before randomization, once deemed eligible, participants reaffirmed their consent to participate on the treatment platform through a brief message.

### Interventions

The guided and unguided IPDT programs were identical, except for the use of therapist support. Guidance was provided during working days and sent within approximately 24 hours after exercises had been completed. Both interventions comprised eight self-help modules delivered over 8 weeks on a secure online platform^35^. The modules contained text and videos, and various exercises that the participants completed online. The aim was to ensure that there were no systematic differences between guided and unguided IPDT, beyond the therapist support itself.

The IPDT programme has been evaluated in two prior RCTs and one smaller feasibility study for adolescent depression^31,36,37^. A treatment based on similar affect-focused principles has also been tested for adults suffering from depression and/or anxiety^38^ and adults with SAD specifically^32^. The treatment programme used in the present trial was adapted from the treatment material used in the treatment of adolescent depression^36^ in order to be better suited for SAD as well as adults. The treatment is text-based, but also contains animated videos and exercises. Participants are encouraged to reflect on and experience underlying emotional conflicts that give rise to and maintain symptoms of social anxiety. The treatment helps participants to monitor and recognize when their anxiety is too high. Anxiety regulation and avoidance of emotions (defenses) are also key components of the treatment. The aim of treatment is to achieve greater insight into the underlying emotional dynamics of the anxiety and decrease emotional avoidance. The final part of the programme contains material on how to identify maladaptive, cyclical relationship patterns and how to communicate and share emotions in key relationships. The complexity of the exercises varies, for instance, some consist of self-ratings of defenses or maladaptive personality configurations. Others are more demanding, such as expressive writing exercises or the implementation of daily routines to improve emotional awareness. The treatment programme is based on Malan’s triangle of conflict^39^, where unconscious emotions are thought to be underlying anxiety. Furthermore, defenses such as projection are thought to exacerbate symptoms of SAD. By increasing the capacity for self-observation whilst also confronting and processing the underlying emotions, participants will both gain insight into maladaptive patterns of relating and symptoms will be reduced.

In the guided arm, each participant was assigned a therapist who provided feedback on completed exercises within 24 hours during weekdays. Furthermore, participants could contact their therapist via text messages with questions about the programme, and receive a response within approximately 24 hours. Feedback typically contained empathic and validating utterances, but also more psychodynamically informed work. For instance, it is common for the therapists to interpret the issues described by the participants in terms of the triangle of conflict, i.e., highlighting how emotions trigger anxiety that is regulated through the use of defenses. Furthermore, it is typical for the therapists to make links between maladaptive relationship patterns as they emerge in different relationships (i.e., the triangle of persons). If participants did not complete any exercises, the therapist sent them a weekly message. Participants in the unguided IPDT were able to contact one of the research coordinators if in need of additional support, for instance if experiencing severe deterioration or an acute crisis, but they were not given therapeutic guidance and had no regular weekly contact.

Participants in the control group were allocated to a waitlist condition. Their symptoms were monitored through weekly symptoms ratings in case of severe deterioration. All participants in the control condition were able to contact one of the research coordinators if needed. However, they were given no regular weekly support or any kind of therapeutic interventions.

### Therapists

The study therapists were 5 Master’s students in their final semester of a clinical psychologist training program. All therapists were specialized in the practice of psychodynamic psychotherapy and had prior experience through their clinical training in treating patients in face-to-face psychodynamic psychotherapy. Therapists had a one-day training in IPDT and received mandatory weekly group supervision of 90 minutes conducted by the treatment developers (JM, KL). Treatment adherence was not systematically monitored, but supervision was based on the written transcripts from feedback on exercises.

### Outcome Measures

All questionnaires were administered via the internet, and each questionnaire was presented one at a time, as suggested by Thorndike et al.^40^. The pre-registered primary outcome measure was symptoms of social anxiety as measured weekly by the the Liebowitz Social Anxiety Scale Self-report (LSAS-SR)^41^. Its relative brevity allows for repeated measurements and research suggests that psychometric properties are maintained also when delivered digitally^42^. The LSAS-SR has been translated to numerous languages and is frequently used in clinical trials rendering it easy to compare results from the present trial with previous clinical trials. LSAS-SR has shown excellent reliability in samples with adults (α = 0.95–0.96) and 12–week test–retest reliability (*r* = 0.83, *p* < 0.01)^41,43^. In the present sample LSAS-SR had an α of .89 at baseline. Results on LSAS-SR have been found to consistently correspond to the clinician-administered version of the same instrument to a high degree^41,44^. A cutoff of ≥60 has been found providing the best balance of sensitivity and specificity for classifying participants with (generalized) SAD^44^, and the instrument has a range of 0–144.

Secondary outcome measures were measured before and after treatment as well as at follow-up. Symptoms of generalized anxiety were measured using the Generalized Anxiety Disorder 7-item scale (GAD-7)^45^. GAD-7 is a brief self-report inventory with a range of 0–21. Higher scores indicate more difficulties regarding generalized anxiety. The GAD-7 demonstrated good internal consistency (α = 0.83) in the present sample at baseline.

Comorbid depression was measured using Patient Health Questionnaire-9 (PHQ-9)^34^, higher scores reflect increased severity of depression (range 0–27). In the present study, no patients with a score ≥20 were included as they were deemed suffering from severe depression indicating a need for treatment of that condition first. PHQ-9 demonstrated acceptable reliability in the present sample (α = 0.70).

Emotion regulation was measured using Difficulties in Emotion Regulation Scale – short form (DERS-16)^46^, a self-report questionnaire capturing global deficiencies in emotion regulation. Higher scores imply greater difficulties in emotion regulation (range 16–80). DERS-16 measures five facets of emotion dysregulation but also presents a global score assessing general emotion dysregulation. In the present study we only use the total score of the questionnaire. DERS-16 demonstrated excellent reliability in the present study sample (α = 0.92).

Self-compassion was assessed using the Self-compassion Scale Short Form (SCS–SF)^47^. The instrument covers four aspects of self-compassion but only the total score was used in the present study (range 12–60). SCS-SF demonstrated good reliability (α = 0.81) at baseline.

Defensive functioning was measured only at pre- and post-treatment with a novel self-report version of the Defensive Mechanism Rating Scale (DMRS-SR30)^48^. DMRS-SR30 consists of items that together represent three factors of defenses of different maturity, it also produces a scale of overall defensive functioning (ODF, range 1–7). In the present study only the total scale of Overall Defensive Functioning was used. The DMRS-SR30 demonstrated good reliability (α = 0.83) in the present sample.

Quality of life was assessed through the use of Brunnsviken Brief Quality of Life Scale (BBQ)^49^. BBQ is a brief self-rating scale validated for use in both clinical and non-clinical samples. It renders a total score of overall life satisfaction, range 0–48. In the present sample, Chronbach’s α was 0.71, indicating acceptable internal consistency.

### Randomization

All participants were randomized simultaneously in one block (1:1:1) by an independent researcher with no involvement in the study, using the computerized randomization tool random.org. They were randomized by using an individual code, meaning that the researcher in charge of randomization did not have access to any personal data about the individual participants. Randomization was conducted after completion of the baseline measures. Since 181 participants were included, as a first step, we randomized which of the three groups would contain one more participant compared to the other two.

### Statistical Methods

Primary analyses were based on all randomly assigned participants (i.e., intent-to-treat analysis [ITT]). In order to fully explore trajectories of change, a multilevel growth curve level strategy was employed using the weekly measurements. Linear mixed-effects models (LMMs) provide unbiased estimates under the missing at random (MAR) assumption, valid if missing observations depend on other observed variables included in the model, i.e., the observed values of the dependent variable or covariates^50^. An LMM with restricted cubic splines was fitted to model change over time. The baseline score was included as a covariate. Linear time was also added as a random effect to allow for a more flexible correlation structure between the repeated measures. All analyses were conducted in R, version 4.3.1^51^, the LMMs were fit using lme4, version 1.1-34^52^. In order to calculate treatment contrasts at each time point and to estimate marginal means, the package *emmeans* version 1.8.8^53^ was used. P-values were adjusted using the Holm–Bonferroni method. All code used in this project together with the individual participant data is available at 10.17605/OSF.IO/CXMTE. To test the robustness of the LMM results, sensitivity analyses were conducted using ANCOVAs. That is, we calculated treatment effects by comparing the groups at posttest while adjusting for pretreatment values of the outcome variable, without making any assumptions about the functional form of the time variable.

Secondary outcomes were not measured weekly, and no follow-up measures were available for the waitlist group since they were crossed over to treatment after the post-treatment assessment. Two models were therefore fitted for each outcome. In the first model, the therapist-guided and self-guided groups were compared using LMMs with 3 measures (post-treatment, 6 months, and 12 months follow-up) while adjusting for the pretest value. Change was modeled as linear, but random intercepts and slopes were included to allow for individual variability. In the second model, post-treatment values were compared using ANCOVA where pretest values were modeled using a restricted cubic spline with 3 knots.

Participants were classified as responders if they exhibited a ≥31% reduction in LSAS-SR scores from pre- to post-treatment. They were considered in remission if they scored ≤ 30 at post-treatment. These cutoffs have previously been used in psychotherapy trials (e.g. refs. ^26,32^.). Deterioration was defined as cases where deterioration reached the threshold for a reliable negative change^21^. Cases where post-treatment assessment was missing were classified as non-responders.

### Power analysis

Power calculations were made before recruitment commenced using Sealed Envelope^54^. For the comparisons between the three conditions (guided IPDT, unguided IPDT and waitlist), 52 participants per group (*n* = 156) would be needed in order to ensure an effect size (Cohen’s *d*) of 0.55 at alpha = 0.05. Assuming a 13% drop-out rate, a total of 180 participants was required. An effect-size of 0.55 should be enough to compare IPDT to WL given earlier research by Johansson et al.^32^ finding a between-group effect size of *d* = 1.05. As no prior studies exist on unguided IPDT the literature on unguided ICBT in the treatment of SAD was consulted. Guo et al.^17^ found an effect size of *g* = 0.68 for unguided ICBT compared to WL. As there was uncertainty whether unguided IPDT would be as effective, we chose a slightly smaller effect of *d* = 0.55 when calculating numbers needed to acquire sufficient power.

## Results

### Descriptive statistics

**Fig. 1:**
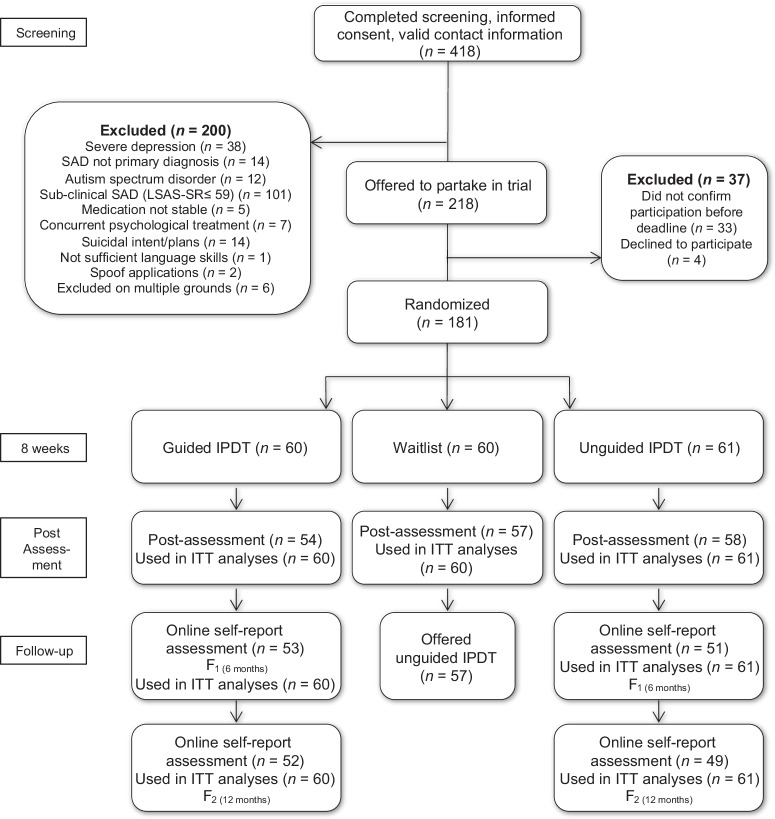
Consort diagram.

The CONSORT diagram for the study is presented in Fig. 1. Between August 2021 and September 2021, a total of 418 potential study participants completed screening; 218 (52%) were found eligible and were thus offered to partake in the trial. Of those offered to partake, 37 either declined participation or refrained from responding, leading to a total sample of 181 being randomized to guided IPDT (*n* = 60), unguided IPDT (*n* = 61) or waitlist (*n* = 60). Demographic data of the sample are summarized in Table 1. Missing data for the primary outcome measure was 23.20% on weekly measures and 6.63% on the post-treatment assessment. At 6 and 12 months after treatment termination, the corresponding numbers were 14.05 and 16.53% on the primary outcome measure. The average participant opened 6.83 modules (SD = 2.27) and 5.59 modules (SD = 2.81) in guided and unguided IPDT respectively, this difference was significant in favor of guided IPDT (*p* = 0.009).

**Table 1:** Demographic data at baseline

Characteristics	Waitlist (*n* = 60)	Unguided IPDT (*n* = 61)	Guided IPDT (*n* = 60)
Gender, *n* (%)			
Female	47 (78.3)	50 (82)	49 (81.7)
Uncertain or other	0 (0)	1 (1.6)	0 (0)
Age (years), mean (SD)	34.6 (11.26)	34.8 (9.94)	35.1 (12.57)
Education, *n* (%)			
Compulsory school	2 (3.3)	2 (3.3)	7 (11.7)
Upper secondary school	23 (38.3)	20 (32.8)	17 (28.3)
University	27 (45)	27 (44.2)	28 (46.7)
Other	8 (13.33)	12 (19.6)	8 (13.4)
Employment status			
Employed	35 (58.3)	34 (55.8)	31 (51.7)
Student	15 (25)	16 (26.2)	14 (23.3)
Unemployed	5 (8.3)	5 (8.2)	7 (11.7)
Parental leave	0 (0)	1 (1.6)	1 (1.7)
Sick leave	3 (5)	1 (1.6)	0 (0)
Other	2 (3.3)	4 (6.6)	7 (11.7)
Currently on psychotropic medication	6 (10)	11 (18)	9 (15)
Earlier experiences of psychotherapy	28 (46.7)	29 (47.5)	34 (56.7)

### Change during treatment

**Fig. 2:**
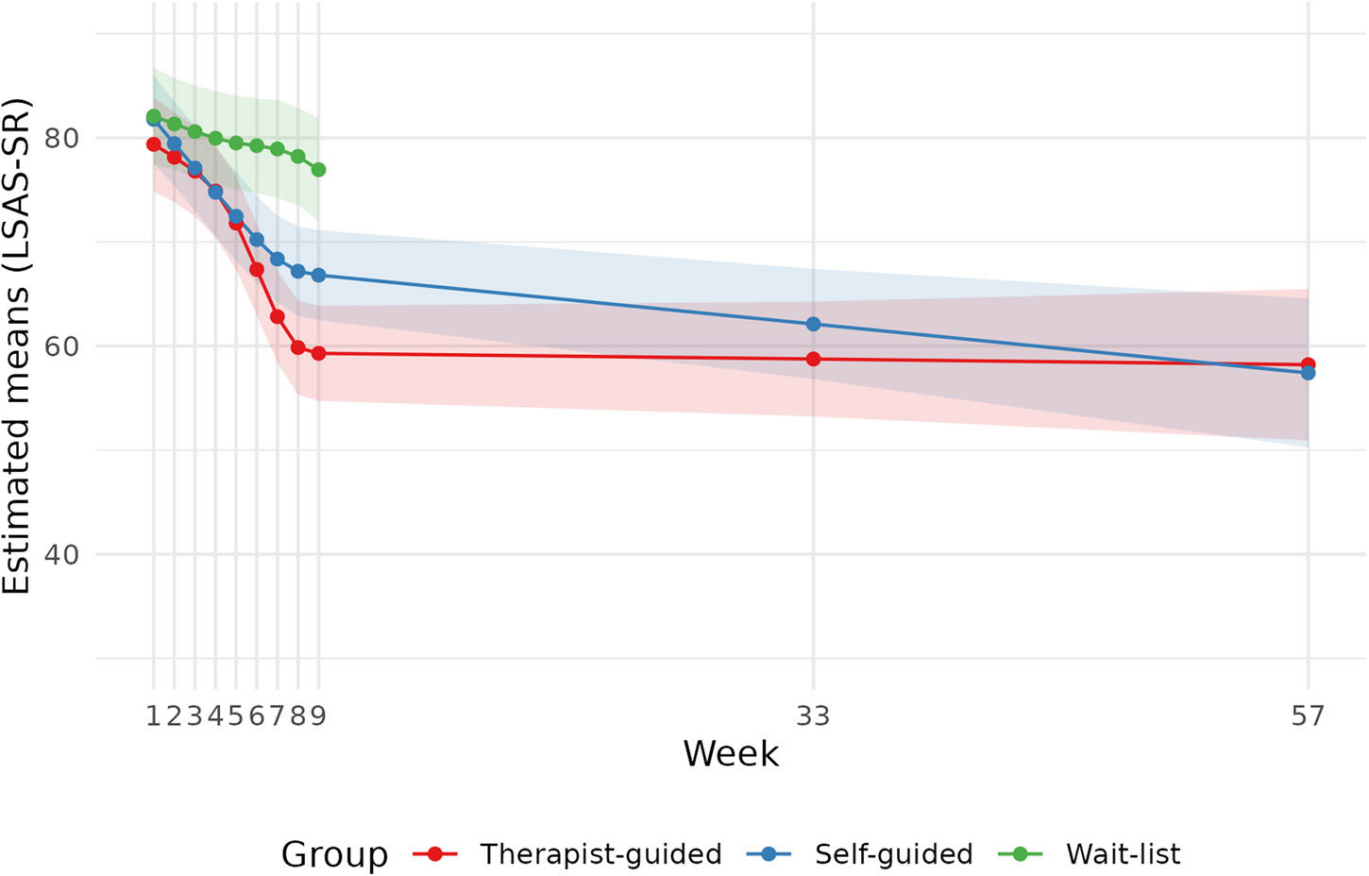
Estimated means at each timepoint.

Social anxiety scores in all three conditions improved during treatment. Estimated marginal means for social anxiety scores over time for all three groups are presented in Fig. 2. At post-treatment, both guided and unguided IPDT were superior to the waitlist condition with guided treatment exhibiting larger between group effects than unguided treatment with a raw score difference of 17.64 (*d* = 1.07 95% CI [0.72, 1.43], *p* < 0.001) and 10.13 (*d* = 0.61, 95% CI [0.25, 0.98], *p* = 0.0018) respectively. In the comparison between guided and unguided IPDT, guided IPDT was found to be associated with a larger improvement with an estimated difference in LSAS-scores at post-treatment of 7.51 (95% CI = 1.85, 13.18). This corresponds to a Cohen’s *d* of 0.46 (95% CI = 0.11, 0.80, *p* = 0.0096). The sensitivity analyses rendered results similar to those of the primary analysis, strengthening the robustness of the results. Since the baseline measure was added as a covariate to the model, within-group changes were calculated from week 1. From week 1 to the post-treatment assessment, the estimated average change in guided IPDT was 20.08 points on LSAS-SR (*d* = 1.22 95% CI [1.07, 1.37], *p* < 0.001). The corresponding change in the unguided IPDT and waitlist was 14.97 (*d* = 0.91 95% [CI 0.75, 1.07], *p* < 0.001) and 5.14 (*d* = 0.31 95% CI [0.12, 0.51], *p* < 0.001).

### Response, remission and deterioration

At end of treatment, 48% (*n* = 29/60) of participants in guided IPDT, 31% (*n* = 19/61) in unguided IPDT and 8% (*n* = 5/60) in the waitlist were classified as responders. Chi square tests indicated that response rates varied significantly across treatment groups (*X*^2^ (2, *N* = 181) = 23.33, *p* < 0.001). Significantly more patients responded to treatment in guided and unguided IPDT as compared to the waitlist condition (*X*^2^ (1, *N* = 120) = 23.64, *p* < .001) and *X*^2^ (1, *N* = 121) = 9.90, *p* = 0.002 respectively). However, the difference between guided and unguided IPDT fell short of statistical significance (*X*^2^(1) = 3.73, *p* = 0.053). Remission was attained for 7% (4/60) and 7% (4/61) in guided and unguided IPDT respectively. In the WL group, 3% (2/60) were classified as remitted. Using Fisher’s exact test to determine if there was a significant association between treatment group and remission rendered a non-significant result (*p* = 0.78). The corresponding numbers regarding reliable deterioration were 0% (0/60) in the guided group, 1.6% (1/61) in the unguided and 10% (6/60) in the waitlist. No adverse events were reported.

### Secondary outcomes

At post-treatment, guided IPDT was significantly superior to waitlist on all secondary outcome measures. Unguided IPDT was significantly superior to waitlist on depressive symptoms and general anxiety, but not on emotion regulation, self-compassion and quality of life. Guided IPDT was significantly superior to unguided PDT on depressive symptoms, with a trend towards superiority for generalized anxiety. See Table 2 for results.

**Table 2:** Differences between groups at post-treatment

Measure	Guided vs Unguided^a^	Guided vs WL^b^	Unguided vs WL^c^
PHQ-9	*d* = 0.43, 95% CI [0.02, 0.84], *p* = 0.04	*d* = 0.85, 95% CI [0.48, 1.21], *p* < 0.001	*d* = 0.37, 95% CI [0.02, 0.73], *p* = 0.04
GAD-7	*d* = 0.34, 95% CI [-0.01, 0.69], *p* = 0.054	*d* = 0.78, 95% CI [0.44, 1.12], *p* < 0.001	*d* = 0.41, 95% CI [0.08, 0.75], *p* = 0.03
DERS-16	*d* = 0.12, 95% CI [-0.19, 0.43], *p* = 0.45	*d* = 0.42, 95% CI [0.13, 0.71], *p* = 0.01	*d* = 0.22, 95% CI [-0.08, 0.51], *p* = 0.29
DMRS-SR30^d^	*d* = 0.28, 95% CI [-0.08, 0.64] *p* = 0.17	*d* = 0.6, 95% CI [0.24, 0.96], p = 0.004	*d* = 0.32, 95% CI [-0.04, 0.68], *p* = 0.17
SCS-SF	*d* = 0.34, 95% CI [-0.08, 0.76], *p* = 0.11	*d* = 0.73, 95% CI [0.36, 1.1], *p* < 0.001	*d* = 0.38, 95% CI [0.02, 0.74], *p* = 0.08
BBQ	*d* = 0.34, 95% CI [-0.03, 0.71], *p* = 0.07	*d* = 0.40, 95% CI [0.08, 0.71], *p* = 0.04	*d* = 0.13, 95% CI [-0.18, 0.44], *p* = 0.42

*PHQ-9* Patient Health Questionnaire-9, *GAD-7* Generalized Anxiety Disorder-7, *DERS-16* Difficulties in Emotion Regulation Scale-16, *DMRS-SR30* Defense Mechanism Rating Scale-Self Rated 30, *SCS* Self-Compassion Scale, *BBQ* Brunnsviken Brief Quality of Life Scale.

^a^Based on LMM analysis. Positive effect sizes indicate superiority of guided treatment.

^b^Based on ANCOVAs. Positive effect sizes indicate superiority of guided treatment.

^c^Based on ANCOVAs. Positive effect sizes indicate superiority of unguided treatment.

^d^Results based on ANCOVAs.

### Follow up

**Fig. 3:**
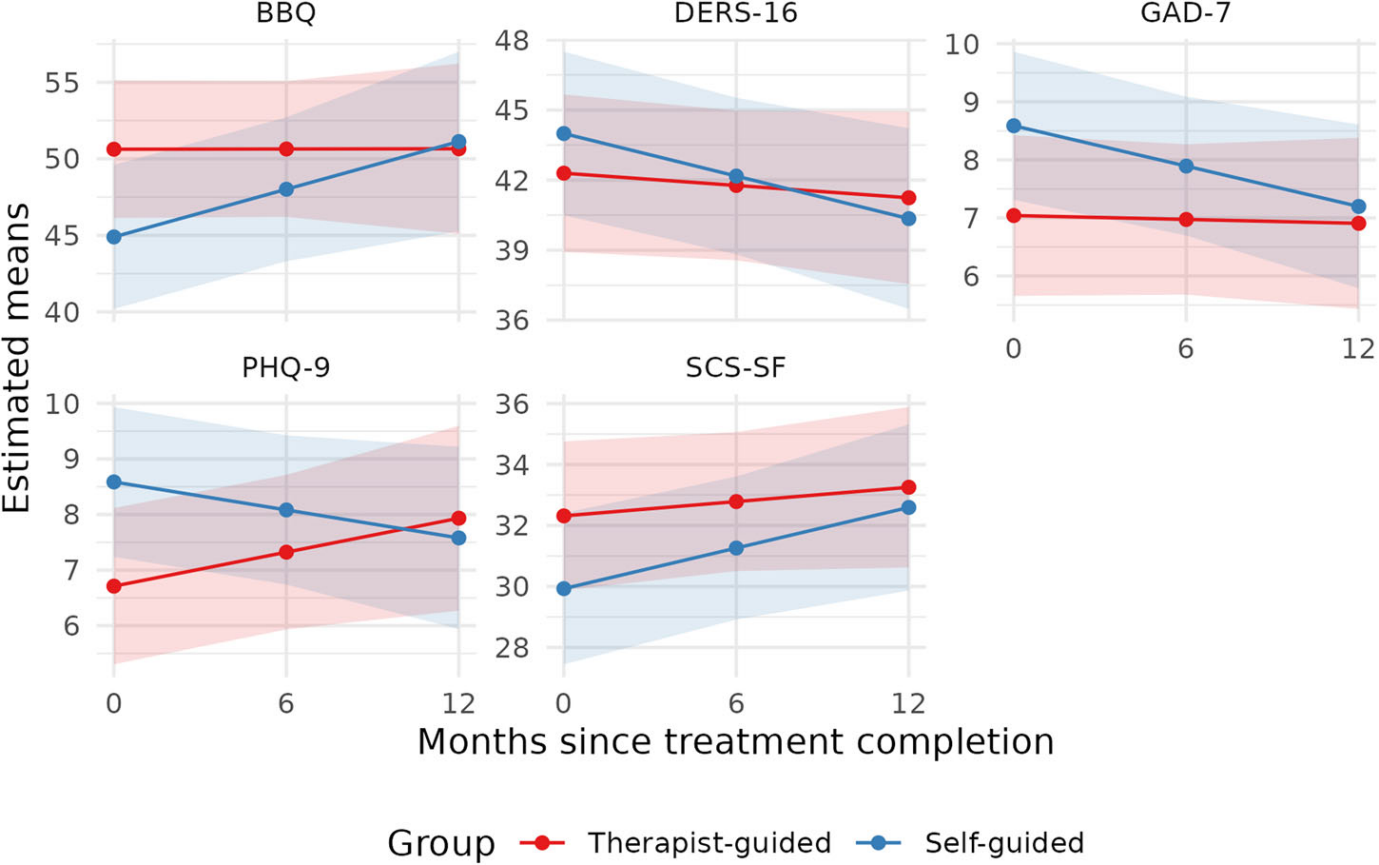
Estimated means on secondary outcomes from post-treatment to follow-up.

From post-treatment to 12-month follow-up, the within-group change for guided IPDT was estimated to a further improvement of 1.08 raw scores on LSAS-SR (*d* = 0.07, 95% CI [−0.24, 0.37], *p* = 0.67). In contrast, the unguided group improved significantly during follow-up, with an estimated change of 9.40 raw scores on LSAS-SR (*d* = 0.57, 95% CI [0.25, 0.88], *p* < 0.001). At twelve months follow-up, there were no longer any significant differences between guided and unguided IPDT on any of the outcome measures. The estimated difference on LSAS-SR was 0.79 points in favor of unguided treatment, corresponding to an effect size of *d* = −0.05. For PHQ-9 the estimated between-group difference was −0.36 points, *d* = −0.08, for GAD-7 0.27 points, *d* = 0.06, for SCS 0.76 points, *d* = 0.11, for BBQ −0.67 points, *d* = −0.04 and for DERS −0.83 points, *d* = −0.06. See Fig. 3 for a graphical illustration of the trajectories of the secondary measures during the follow-up period.

## Discussion

This is the first RCT evaluating guided and unguided IPDT in SAD. Results suggest that, in line with our first hypothesis, both guided and unguided IPDT were superior to a waitlist condition with large and moderate effects on SAD symtoms, respectively. Furthermore, guided IPDT was significantly superior to unguided IPDT at the post-treatment assessment with a small to moderate between-group effect, confirming our second hypothesis. Hypothesis 3, which postulated that both IPDT treatments would be significantly superior to waitlist on secondary outcomes, was only partly supported. Guided IPDT was significantly superior compared to waitlist on all secondary outcomes, with moderate to large effects on comorbid depression, generalized anxiety, defensive functioning and self-compassion and small effects on quality of life and emotion regulation. However, effects for unguided IPDT were less pronounced with only decreases in depression and generalized anxiety reaching statistical significance compared to waitlist. Hypothesis 4 was also partly supported, as guided IPDT was superior to unguided IPDT in the treatment of comorbid symptoms of depression. Differences between the two active conditions on other secondary outcomes were non-significant, with a trend towards significant superiority for guided IPDT on comorbid anxiety. The results on comorbid depression in favor of guided IPDT are especially noteworthy since comorbidity is frequent and associated with adverse outcomes such as higher risk of relapse and decreased functionality^55^.

The fact that guided treatment was associated with significantly larger effects has been previously shown for depression^56^ but is in contrast with other trials for patients with SAD^57–59^. However, in one of the aforemention trials, made by Furmark et al.^57^, effects for guided ICBT compared to waitlist were in line with those for unguided PDT in this trial.

At end of treatment, roughly half of all participants in guided IPDT were classified as responders. This seems comparable to the 58% response rate found in the study on IPDT by Johansson et al.^32^ and to the response rate (52%) found in the largest RCT of face-to-face PDT for SAD^26^. However, it should be noted that Leichsenring et al.^26^ used the observer-rated version of LSAS and that samples may differ between the respective studies. As expected, fewer participants (31%) were classified as responders in the unguided treatment. No patients deteriorated reliably in guided IPDT and only one patient deteriorated reliably in unguided IPDT, indicating that both treatments seem to be safe in terms of iatrogenic effects. Compared to the findings from Rozental et al.^60^, reporting deterioration rates of 5.8% in internet delivered CBT treatments and 17.4% in control conditions, the deterioration rates observed in this study are notably low. Still, this should be further investigated using broader sets of questions such as the Negative Effects Questionnaire^61^. No adverse events were reported and no patients were withdrawn from the study.

When assessing follow up data, both active treatments yielded similar results; guided and unguided IPDT converged over the follow-up period and at the 12-months follow-up the difference was almost nonexistent. The same pattern was evident also on secondary outcomes, where all between-group differences were negligible and non-significant. For most measures, this seems to be due to a sleeper effect in the unguided group, meaning that this group displayed continuous improvements over the follow-up period, whilst the group having received guided IPDT remained stable. The exception from this pattern is depressive symptoms, measured with PHQ-9, where the unguided group continued to improve further, while the guided group also displays a deterioration, meaning that their slopes cross at the 12-month follow-up point. The pattern for social anxiety, general anxiety, self-compassion and quality of life, where participants in the unguided group exhibited less improvements at post-treatment, but “caught up” during the follow-up period is interesting. It could be hypothesized that the guided treatment led to faster improvements, but that those improvements made in the unguided group still led to positive cycles, facilitating further positive change. Interestingly, a recent study by Hagberg et al.^62^ on internet-delivered assertiveness training also found no significant differences between guided and unguided interventions in their impact on participants at follow-up, further corroborating the findings of this study. However, it should be noted that there was no control group during the follow up-period, meaning that there is no way to control for spontaneous remission. Additionally, it has been argued that unguided internet-delivered treatments make less sense when based on psychodynamic theory, given the centrality of the therapeutic relationship in PDT^63^. Results from the present trial seem to corroborate this to some extent, given the added benefit of guidance at treatment termination. However, mechanisms of change should be investigated further to deepen our understanding of treatment effects in both active conditions.

The results from the present study also allow for tentative comparisons to ICBT. Guo et al.^17^ found that guided ICBT was superior to waitlist conditions (*g* = –0.81). Results from the present study and Johansson et al.^32^ suggest that IPDT might be of similar efficacy since both studies have rendered large between group effect sizes (*d* = *–*1.07 and –1.05 respectively). It is worth noting that Clark et al.^64^ tested ICBT and reported an exceptionally high effect size of *d* = 2.2. To our knowledge, this is the first study that tested an unguided IPDT intervention, but the results are again similar to those obtained by ICBT^17^. Unguided IPDT showcased a medium effect compared to the waitlist condition (*d* = –0.61) which could be compared to unguided ICBT *g* = –0.68. Again, these comparisons are tentative and it should be noted that the number of studies on ICBT by far surpasses the two studies on IPDT for SAD. Still, results from the present trial together with results from Johansson et al.^32^ should warrant larger, comparative studies where the two treatments are directly compared to each other. Furthermore, different treatments might suit different patients – this is also an area in need of further investigation. Future research should delve deeper into the effects of therapist participation, potentially augmented by AI (as suggested by Seiferth et al.^65^), and how it interacts with individual participant characteristics.

Although we found promising results from two relatively short treatments, the majority of participants in either treatment condition did not remit during treatment. This indicates that 8 weeks of treatment could be too short for a sample with such high symptom burden at start of treatment. Interestingly, a systematic review by Scholten et al.^66^ found that higher baseline severity of social anxiety symptoms was associated with larger symptom reductions after CBT, suggesting that the magnitude of treatment effects may be influenced by the initial symptom burden. Compared to other studies on PDT and IPDT, fewer patients remitted^26,32^, but it should be noted that the present study utilized a substantially higher cut-off for inclusion (≥60 as compared to ≥ 30). In order to reach remission, patients had to at least improve ≥30 points over 8 weeks of treatment. In fact, the average patient entered treatment with scores above 80, meaning that even larger improvements would have been needed. As only 7% reached remission in both active conditions this clearly indicates the need for a longer or more intense treatment. Of course, it is also possible that IPDT would have been insufficient for reaching remission even if it had been provided over a longer period of time. On the other hand, as can be seen from Fig. 2, change over time is substantial and effects are maintained (or in the context of unguided treatment, further improved) over 6- and 12-month follow-up. This suggests that even if patients did not remit, effects of treatment were robust and stable over time. This is noteworthy since scores at intake suggest that participants in the present trial suffered from a high symptom burden. It should also be noted that a recent investigation into the psychometric properties of LSAS-SR indicates that cutoffs for response and remission might be somewhat conservative, leading to suggestions of new cutoffs^67^. Adhering to these slightly altered cutoffs would have minimally increased the number of responders and remitters, but would also have made it more difficult to compare results from the present trial to previous studies.

Nationwide recruitment and relatively low thresholds for inclusion (i.e., patients were able to be included regardless of previous treatment experiences, duration of SAD, preference for internet-delivered treatment, suitability and motivation for psychological treatment) could all potentially increase the generalizability of the results. Lindner et al.^68^ found that participants recruited through sources such as social media and online advertisements presented with more severe depression and anxiety compared to those from more passive sources, highlighting the potential influence of recruitment source on the clinical characteristics of participants in online treatments. The study was sufficiently powered to detect medium differences between conditions, comparisons based on smaller estimated differences suffered from lack of power. To date, this is also one of the larger studies conducted on IPDT^30^. Weekly measurements of the primary outcome and a relatively high amount of completed data are also to be considered strengths of the study.

Limitations of the study include the lack of a diagnostic assessment at baseline and at post-treatment. Although established cutoffs were used to include participants with a high likelihood for fulfilling the diagnostic criteria for SAD^44^ a diagnostic interview would have strengthened the validity of the diagnosis^67^. However, the number of participants with prior experiences from psychotherapy (roughly 50%) suggests that this was indeed a sample with longstanding and debilitating psychiatric problems. Another limitation is the lack of observer-rated outcome measures and blinded assessments. Even though preference for internet-delivered treatment was not assessed, the fact that participants were self-referred might have led to selection-bias with a sample more positively inclined towards remotely delivered treatment. This could lead to reduced generalizability in terms of effects and attrition if IPDT was to be implemented in healthcare and offered to patients who were not self-referred. It should also be noted that all of the study therapists were relatively inexperienced Master’s students, yet to become licensed psychologists. It is possible that licensed psychologists / psychotherapists with extensive clinical experience would have produced different results. However, evidence from ICBT in SAD suggests that experience in therapists does not affect outcomes^17,69^. All therapists were supervised by experienced psychologists and treatment developers, with supervision based on transcripts from the treatment. This should decrease the risks for therapist drift, but it should be noted that the integrity of IPDT could have been furthered by using independent, blinded raters of treatment adherence. At the same time, this is rarely done in studies on internet-delivered treatments as a substantial part of the treatment consists of standardized self-help material.

The average participant opened 6.83 modules (SD = 2.27) and 5.59 modules (SD = 2.81) in guided and unguided IPDT respectively. However, we do not have any data on how much time participants spent in the respective modules. This kind of data would of course be highly interesting, but given the fact that they are allowed to also download the material, that is not possible to measure.

Finally, research clearly shows that waitlist controls are not a very strict test for treatment efficacy since the effects of waiting for treatment on SAD are negligible *g* = 0.13^70^. Utilizing diverse control conditions, as emphasized by Goldberg et al.^71^, can provide a more comprehensive understanding of the strength and nuances of the evidence. Further trials should compare guided and unguided IPDT to more active control conditions. On the other hand, guided IPDT’s superiority as compared to unguided IPDT at post-treatment could be seen as a more stringent test of efficacy.

In conclusion, results in the present trial largely corroborate earlier findings from Johansson et al.^32^, and the support for IPDT specifically and PDT in general, in the treatment of SAD is further strengthened. Although both guided and unguided treatment were associated with improvements compared to a waiting list, patients in guided IPDT improved more rapidly. Given the promising results found in two RCTs, IPDT should be compared to other bonafide internet-delivered therapies for SAD, such as ICBT in adequately powered trials. Preferably, such a trial could utilize a non-inferiority design to confirm whether IPDT is as effective as ICBT.

## Data Availability

All data used in this project together with the individual participant data is available at 10.17605/OSF.IO/CXMTE.

## Code availability

All code used in this project is available at 10.17605/OSF.IO/CXMTE.

## References

[1] American Psychiatric Association. *Diagnostic and Statistical Manual of Mental Disorders*. (American Psychiatric Publishing, Washington, DC, 2013).

[2] WHO World Mental Health Survey Collaborators. (2017). The cross-national epidemiology of social anxiety disorder: Data from the World Mental Health Survey Initiative. BMC Med..

[3] Arad G, Shamai-Leshem D, Bar-Haim Y (2021). Social distancing during a COVID-19 lockdown contributes to the maintenance of social anxiety: a natural experiment. Cogn. Ther. Res..

[4] Kindred R, Bates GW (2023). The influence of the COVID-19 pandemic on social anxiety: a systematic review. IJERPH.

[5] Mayo-Wilson E (2014). Psychological and pharmacological interventions for social anxiety disorder in adults: a systematic review and network meta-analysis. Lancet Psychiatry.

[6] Narr RK, Teachman BA (2017). Using advances from cognitive behavioral models of anxiety to guide treatment for social anxiety disorder: using advances from CBM to treat social anxiety disorder. J. Clin. Psychol..

[7] Cuijpers P, Cristea IA, Karyotaki E, Reijnders M, Huibers MJH (2016). How effective are cognitive behavior therapies for major depression and anxiety disorders? A meta-analytic update of the evidence. World Psychiatry.

[8] Kindred R, Bates GW, McBride NL (2022). Long-term outcomes of cognitive behavioural therapy for social anxiety disorder: A meta-analysis of randomised controlled trials. J. Anxiety Disord..

[9] van Dis EAM (2020). Long-term outcomes of cognitive behavioral therapy for anxiety-related disorders: a systematic review and meta-analysis. JAMA Psychiatry.

[10] Alonso J (2018). Treatment gap for anxiety disorders is global: Results of the World Mental Health Surveys in 21 countries. Depress. Anxiety.

[11] Wang PS (2005). Twelve-month use of mental health services in the United States: Results from the national comorbidity survey replication. Arch. Gen. Psychiatry.

[12] Olatunji BO, Cisler JM, Tolin DF (2007). Quality of life in the anxiety disorders: A meta-analytic review. Clin. Psychol. Rev..

[13] Acarturk C (2009). Economic costs of social phobia: A population-based study. J. Affect. Disord..

[14] Goetter EM (2020). Barriers to mental health treatment among individuals with social anxiety disorder and generalized anxiety disorder. Psychol. Serv..

[15] Smith KA (2023). Digital mental health: challenges and next steps. BMJ Ment. Health.

[16] Hedman E (2011). Internet-based cognitive behavior therapy vs. cognitive behavioral group therapy for social anxiety disorder: a randomized controlled non-inferiority trial. PLoS ONE.

[17] Guo S (2021). The efficacy of internet-based cognitive behavioural therapy for social anxiety disorder: A systematic review and meta-analysis. Clin. Psychol. Psychother..

[18] Winter, H. R., Norton, A. R., Burley, J. L. & Wootton, B. M. Remote cognitive behaviour therapy for social anxiety disorder: a meta-analysis. *J. Anxiety Disord.* 102787 (2023) 10.1016/j.janxdis.2023.102787. 10.1016/j.janxdis.2023.102787 37890219

[19] Hofmann SG, Bögels SM (2006). Recent advances in the treatment of social phobia: introduction to the special issue. J. Cogn. Psychother..

[20] Leichsenring F, Leweke F (2017). Social anxiety disorder. N. Engl. J. Med.

[21] Jacobson NS, Truax P (1991). Clinical significance: a statistical approach to defining meaningful change in psychotherapy research. J. Consult. Clin. Psychol.

[22] Boettcher J, Carlbring P, Renneberg B, Berger T (2013). Internet-based interventions for social anxiety disorder - an overview. Verhaltenstherapie.

[23] McHugh RK, Whitton SW, Peckham AD, Welge JA, Otto MW (2013). Patient preference for psychological vs pharmacologic treatment of psychiatric disorders: a meta-analytic review. J. Clin. Psychiatry.

[24] Keefe JR, McCarthy KS, Dinger U, Zilcha-Mano S, Barber JP (2014). A meta-analytic review of psychodynamic therapies for anxiety disorders. Clin. Psychol. Rev..

[25] Lilliengren P (2023). A comprehensive overview of randomized controlled trials of psychodynamic psychotherapies. Psychoanal. Psychother..

[26] Leichsenring F (2013). Psychodynamic therapy and cognitive-behavioral therapy in social anxiety disorder: a multicenter randomized controlled trial. AJP.

[27] Leichsenring F (2014). Long-term outcome of psychodynamic therapy and cognitive-behavioral therapy in social anxiety disorder. AJP.

[28] Salzer S (2018). Cognitive-behavioral and psychodynamic therapy in adolescents with social anxiety disorder: a multicenter randomized controlled trial. Psychother. Psychosom..

[29] Bögels SM, Wijts P, Oort FJ, Sallaerts SJM (2014). Psychodynamic Psychotherapy versus cognitive behavior therapy for social anxiety disorder: an efficacy and partial effectiveness trial: PDT or CBT for social anxiety disorder. Depress. Anxiety.

[30] Lindegaard T, Berg M, Andersson G (2020). Efficacy of internet-delivered psychodynamic therapy: systematic review and meta-analysis. Psychodyn. Psychiatry.

[31] Mechler J (2022). Therapist-guided internet-based psychodynamic therapy versus cognitive behavioural therapy for adolescent depression in Sweden: a randomised, clinical, non-inferiority trial. Lancet Digit.Health.

[32] Johansson R (2017). Internet-based affect-focused psychodynamic therapy for social anxiety disorder: A randomized controlled trial with 2-year follow-up. Psychotherapy.

[33] Posner K (2011). The Columbia-Suicide Severity Rating Scale: Initial Validity and Internal Consistency Findings From Three Multisite Studies With Adolescents and Adults. Am. J. Psychiatry.

[34] Kroenke K, Spitzer RL, Williams JBW (2001). The PHQ-9: Validity of a brief depression severity measure. J. Gen. Intern. Med..

[35] Vlaescu G, Alasjö A, Miloff A, Carlbring P, Andersson G (2016). Features and functionality of the Iterapi platform for internet-based psychological treatment. Internet Interven..

[36] Lindqvist K (2020). Affect-focused psychodynamic internet-based therapy for adolescent depression: randomized controlled trial. J. Med. Internet Res..

[37] Midgley N (2021). The depression: Online Therapy Study (D:OTS)—A pilot study of an internet-based psychodynamic treatment for adolescents with low mood in the UK, in the context of the COVID-19 pandemic. Int. J. Environ. Res. Public Health.

[38] Johansson R (2013). Affect-focused psychodynamic psychotherapy for depression and anxiety through the Internet: a randomized controlled trial. PeerJ.

[39] Malan, D. H. *Individual Psychotherapy and the Science of Psychodynamics*. (Butterworths, London; Boston, 1995).

[40] Thorndike FP (2009). Web-based measurement: Effect of completing single or multiple items per webpage. Comput. Hum. Behav..

[41] Fresco DM (2001). The Liebowitz Social Anxiety Scale: a comparison of the psychometric properties of self-report and clinician-administered formats. Psychol. Med..

[42] Hedman E (2010). Internet administration of self-report measures commonly used in research on social anxiety disorder: A psychometric evaluation. Comput. Hum. Behav..

[43] Baker SL, Heinrichs N, Kim H-J, Hofmann SG (2002). The Liebowitz social anxiety scale as a self-report instrument: a preliminary psychometric analysis. Behav. Res. Ther.

[44] Rytwinski NK (2009). Screening for social anxiety disorder with the self-report version of the Liebowitz Social Anxiety Scale. Depress. Anxiety.

[45] Spitzer RL, Kroenke K, Williams JBW, Löwe B (2006). A Brief measure for assessing generalized anxiety disorder: The GAD-7. Arch. Intern. Med..

[46] Bjureberg J (2016). Development and validation of a brief version of the difficulties in emotion regulation scale: The DERS-16. J. Psychopathol. Behav. Assess..

[47] Raes F, Pommier E, Neff KD, Van Gucht D (2011). Construction and factorial validation of a short form of the Self-Compassion Scale. Clin. Psychol. Psychother..

[48] Di Giuseppe M (2020). Preliminary reliability and validity of the DMRS-SR-30, a novel self-report measure based on the defense mechanisms rating scales. Front. Psychiatry.

[49] Lindner P (2016). The Brunnsviken Brief Quality of Life Scale (BBQ): Development and psychometric evaluation. Cogn. Behav. Ther..

[50] Little RJA (1995). Modeling the drop-out mechanism in repeated-measures studies. J. Am. Stat. Assoc..

[51] R Core Team. R: A language and environment for statistical computing. R Foundation for Statistical Computing, Vienna, Austria. https://www.R-project.org/ (2023).

[52] Bates D, Mächler M, Bolker B, Walker S (2015). Fitting Linear Mixed-Effects Models Using lme4. J. Stat. Soft.

[53] Lenth R. emmeans: Estimated Marginal Means, aka Least-Squares Means. R package version 1.7.4-1. https://CRAN.R-project.org/package=emmeans (2023).

[54] Sealed Envelope Ltd. 2012. Power calculator for continuous outcome superiority trial. [Online]. https://www.sealedenvelope.com/power/continuous-superiority/ (2012).

[55] Koyuncu A, İnce E, Ertekin E, Tükel R (2019). Comorbidity in social anxiety disorder: diagnostic and therapeutic challenges. DIC.

[56] Moshe I (2021). Digital interventions for the treatment of depression: A meta-analytic review. Psychol. Bull..

[57] Furmark T (2009). Guided and unguided self-help for social anxiety disorder: randomised controlled trial. Br. J. Psychiatry.

[58] Dear BF (2018). Treating anxiety and depression in young adults: A randomised controlled trial comparing clinician-guided versus self-guided Internet-delivered cognitive behavioural therapy. Aust. N. Z. J. Psychiatry.

[59] Fogliati VJ (2016). Disorder-specific versus transdiagnostic and clinician-guided versus self-guided internet-delivered treatment for panic disorder and comorbid disorders: A randomized controlled trial. J. Anxiety Disord..

[60] Rozental A, Magnusson K, Boettcher J, Andersson G, Carlbring P (2017). For better or worse: An individual patient data meta-analysis of deterioration among participants receiving Internet-based cognitive behavior therapy. J. Consult. Clin. Psychol..

[61] Rozental A, Kottorp A, Boettcher J, Andersson G, Carlbring P (2016). Negative effects of psychological treatments: an exploratory factor analysis of the negative effects questionnaire for monitoring and reporting adverse and unwanted events. PLoS ONE.

[62] Hagberg T (2023). Efficacy of transdiagnostic cognitive-behavioral therapy for assertiveness: A randomized controlled trial. Internet Interven..

[63] Maroti D, Hallberg H, Lindqvist K, Mechler J (2022). Using psychodynamic principles in guided internet-delivered therapy (IPDT). Psychoanal. Psychother..

[64] Clark DM (2023). More than doubling the clinical benefit of each hour of therapist time: a randomised controlled trial of internet cognitive therapy for social anxiety disorder. Psychol. Med..

[65] Seiferth C (2023). How to e-mental health: a guideline for researchers and practitioners using digital technology in the context of mental health. Nat. Ment. Health.

[66] Scholten W (2023). Baseline severity as a moderator of the waiting list–controlled association of cognitive behavioral therapy with symptom change in social anxiety disorder: a systematic review and individual patient data meta-analysis. JAMA Psychiatry.

[67] von Glischinski M (2018). Liebowitz Social Anxiety Scale (LSAS): Optimal cut points for remission and response in a German sample. Clin. Psychol. Psychother..

[68] Lindner P, Nyström MBT, Hassmén P, Andersson G, Carlbring P (2015). Who seeks ICBT for depression and how do they get there? Effects of recruitment source on patient demographics and clinical characteristics. Internet Interven..

[69] Andersson G, Carlbring P, Furmark T, on behalf of the S. O. F. I. E. Research Group (2012). Therapist experience and knowledge acquisition in internet-delivered CBT for social anxiety disorder: a randomized controlled trial. PLoS ONE.

[70] Steinert C, Stadter K, Stark R, Leichsenring F (2017). The effects of waiting for treatment: a meta-analysis of Waitlist Control Groups in randomized controlled trials for social anxiety disorder: the effects of waiting for treatment. Clin. Psychol. Psychother..

[71] Goldberg SB, Sun S, Carlbring P, Torous J (2023). Selecting and describing control conditions in mobile health randomized controlled trials: a proposed typology. npj Digit. Med..

